# Effects of ischemic conditioning on head and neck free flap oxygenation: a randomized controlled trial

**DOI:** 10.1038/s41598-022-12374-3

**Published:** 2022-05-17

**Authors:** Se-Hee Min, Suk Hyung Choe, Won Shik Kim, Soon-Hyun Ahn, Youn Joung Cho

**Affiliations:** 1 Department of Anesthesiology and Pain Medicine, Seoul National University Hospital, Seoul National University College of Medicine, 101 Daehak-ro, Jongno-gu, Seoul, 03080 South Korea; 2 Department of Anesthesiology and Pain Medicine, Chung-Ang University College of Medicine, 102 Heukseok-ro, Heukseok-dong, Dongjak-gu, Seoul, 06973 South Korea; 3 Department of Otorhinolaryngology-Head and Neck Surgery, Seoul National University Hospital, Seoul National University College of Medicine, 101 Daehak-ro, Jongno-gu, Seoul, 03080 South Korea; 4 Jeil ENT Clinic, 23, Nonhyeon-ro 131-gil, Gangnam-gu, Seoul, 06045 South Korea

**Keywords:** Randomized controlled trials, Head and neck cancer

## Abstract

Flap failure after microvascular reconstructive surgery is a rare but devastating complication caused by reperfusion injury and tissue hypoperfusion. Remote ischemic conditioning (RIC) provides protection against ischemia/reperfusion injury and reduces tissue infarction. We hypothesized that RIC would enhance flap oxygenation and exert organ-protective effects during head and neck free flap reconstructive surgery. Adult patients undergoing free flap transfer surgery for head and neck cancer were randomized to receive either RIC or sham-RIC during surgery. RIC consisted of four cycles of 5-min ischemia and 5-min reperfusion applied to the upper or lower extremity. The primary endpoint, tissue oxygen saturation of the flap, was measured by near-infrared spectroscopy on the first postoperative day. Organ-protective effects of RIC were evaluated with infarct size of rat hearts perfused with plasma dialysate from patients received RIC or sham-RIC. Between April 2018 and July 2019, 50 patients were randomized (each n = 25) and 46 were analyzed in the RIC (n = 23) or sham-RIC (n = 23) groups. Tissue oxygen saturation of the flap was similar between the groups (85 ± 12% vs 83 ± 9% in the RIC vs sham-RIC groups; *P* = 0.471). Myocardial infarct size after treatment of plasma dialysate was significantly reduced in the RIC group (44 ± 7% to 26 ± 6%; *P* = 0.018) compared to the sham-RIC group (42 ± 6% to 37 ± 7%; *P* = 0.388). RIC did not improve tissue oxygenation of the transferred free flap in head and neck cancer reconstructive surgery. However, there was evidence of organ-protective effects of RIC in experimental models.

Trial registration: Registry number of ClinicalTrials.gov: NCT03474952.

## Introduction

Microvascular free tissue transfer is a treatment of choice for reconstruction during head and neck cancer surgery^1^. Although the overall success rate is reported to be > 90%^2,3^, partial or total flap loss is still a major complication and its incidence is reported as 1–5% and 7–20%, respectively^4^. The main causes of flap loss are insufficient tissue perfusion and oxygenation, and ischemia/reperfusion (I/R) injury resulting in tissue ischemia and necrosis^4^.

Remote ischemic conditioning (RIC) provides organ protection against I/R injury by enhancing tissue tolerance to ischemia. RIC involves brief cycles of non-lethal ischemia and reperfusion of the remote organs to protect major target tissue, such as lung, myocardium, or kidney^5–7^. Previous studies showed that RIC enhanced tissue microcirculation in muscle flaps subjected to prolonged ischemia in rats^8,9^. In another study, brief cycles of RIC improved cutaneous microcirculation of the contralateral thigh^10^. However, there is a lack of research on the effects of RIC during head and neck free flap reconstruction.

We hypothesized that RIC would improve flap tissue perfusion and exert organ-protective effects in head and neck cancer patients. To evaluate our hypothesis, we conducted a randomized clinical trial to provide either RIC or sham-RIC during free flap reconstruction and performed an animal experiment using a plasma dialysate in an ex vivo I/R injury model.

## Methods

### Study design and ethical approval

This prospective randomized controlled trial was approved by the Institutional Review Board of Seoul National University Hospital (08/03/2018, #1802-027-920) and was registered at clinicaltrials.gov (23/03/2018, NCT03474952). The study was conducted in accordance with Good Clinical Practice guidelines and the Declaration of Helsinki. All animal experiments were approved by the Institutional Animal Care and Use Committee (IACUC) of Seoul National University (SNU-180515-2) and performed following the ARRIVE guidelines and the Guide for the Care and Use of Laboratory Animals published by the US National Institutes of Health (NIH, Bethesda, MD, USA). Informed consent was obtained from all participants and could be withdrawn at any time.

### Study population and randomization

Patients aged ≥ 20 years, undergoing head and neck free flap reconstructive surgery in a single tertiary academic center were included. The exclusion criteria were body mass index < 18 or > 35 kg/m^2^, uncontrolled diabetes, beta-blocker use^11^, arterio-venous fistulas or anomalies of the extremities, peripheral vasculopathies or neuropathies^12^, coagulopathies, history of radiation therapy, and refusal to participate. Use of beta-blocker was involved in exclusion criteria as it has been shown to attenuate the effects of ischemic conditioning^11^. Neuropathies were diagnosed according to clinical symptoms, clinical exmination, and electrophysiological sensory testing by neurologists in symptomatic patients.

**Figure 1:**
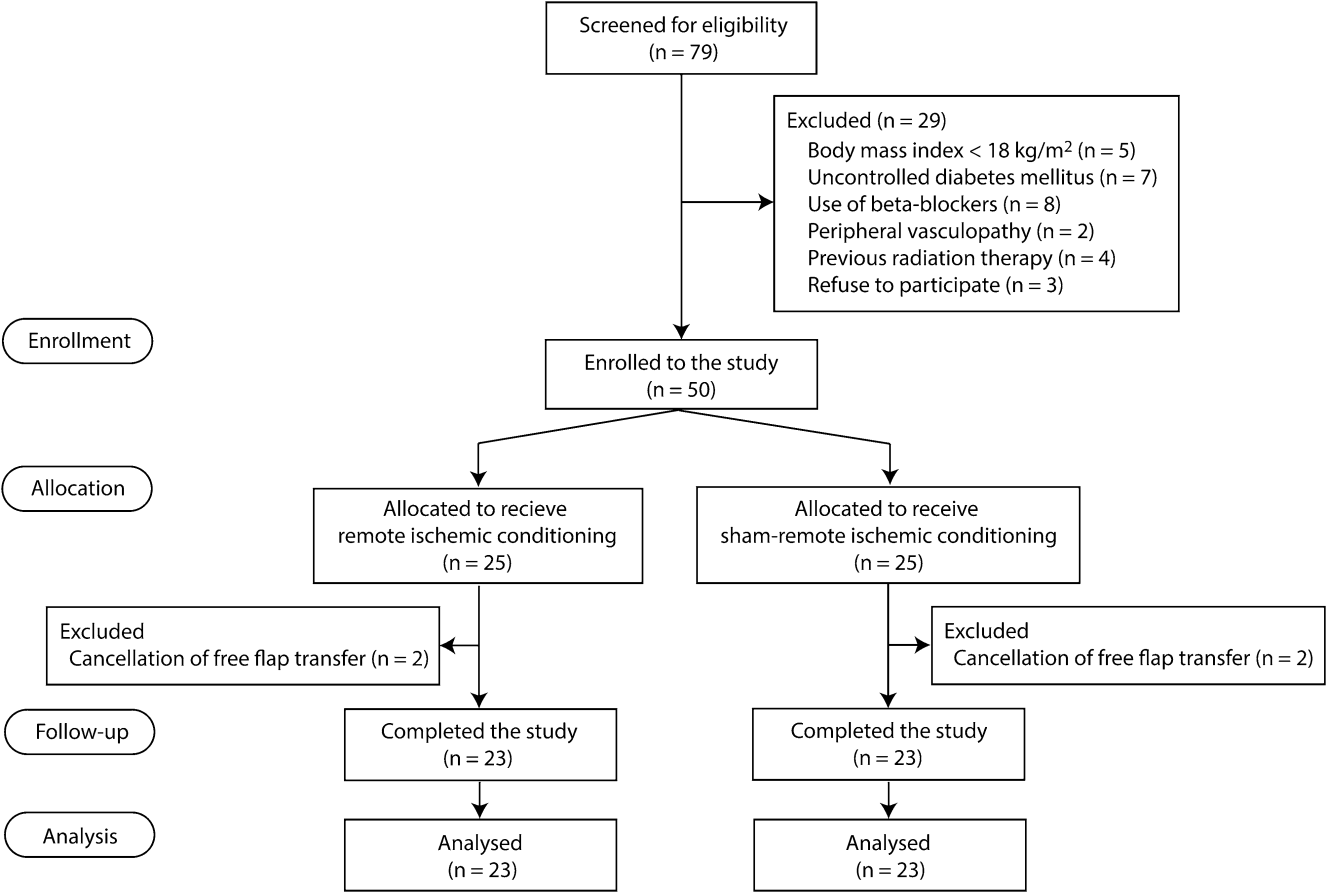
CONSORT diagram of patient recruitment.

After obtaining written informed consent, patients were randomized to receive either RIC or sham-RIC (Fig. 1). Block randomization (blocks of four) for a 1:1 patient allocation ratio was performed using a computer-generated randomization program by an independent research nurse. We also used a random five-number table from a computer-generated program to randomly select five patients to obtain serum dialysate for animal experiments in each group. Group assignments were concealed in an opaque envelop, and all investigators, surgeons, and patients were blinded to the group allocation.

### Study protocol

Non-invasive blood pressure, pulse oximetry, and electrocardiography were monitored. The temperature of the operating room was maintained at 22 ± 1 °C. After preoxygenation, general anesthesia was induced by intravenous administration of propofol (2 mg/kg) with target-controlled infusion of remifentanil (effect-site concentration: 2–4 ng/mL). Rocuronium (0.6 mg/kg) was injected for neuromuscular blockade, the trachea was intubated, and the lungs were ventilated in volume-controlled mode with a tidal volume of 6–8 mL/kg, positive end-expiratory pressure of 5 cm H_2_O, and a fraction of inspired oxygen of 40–50%. Anesthesia was maintained with inhalation of 1.5–2.5 vol% of sevoflurane.

All operative procedures were performed by two experienced head and neck surgeons (S.-H.A. and W.S.K.). Following wide excision of the tumor with free resection margins, the anterolateral thigh, radial forearm, latissimus dorsi, or fibula was selected as the donor site for free tissue transfer. After the vascular pedicle had been identified, the flap was developed and applied to the defect site. The arteries and veins were anastomosed, and primary wound closure was performed after elevation of the flap.

**Figure 2:**
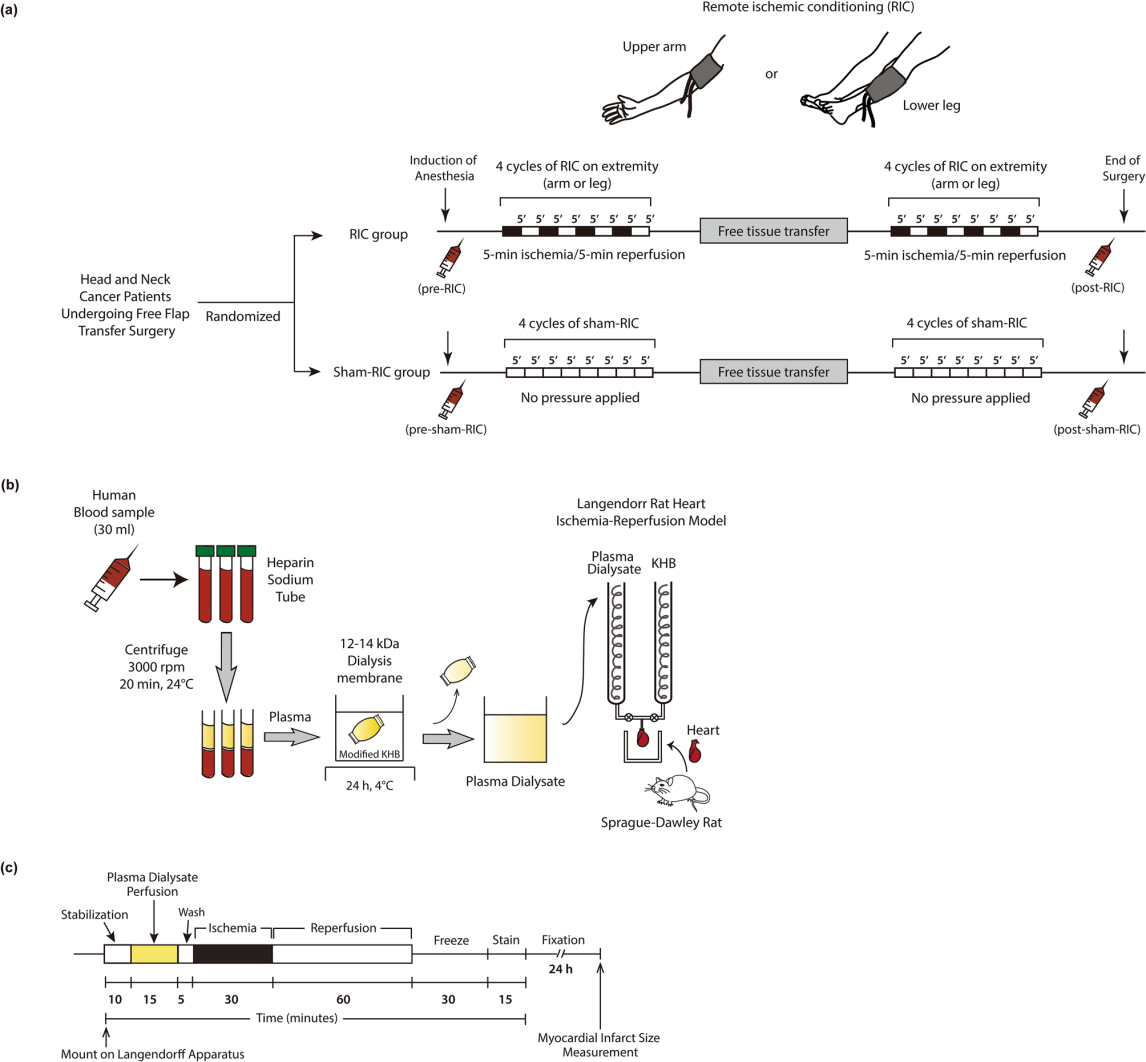
Study protocol of remote ischemic preconditioning or sham in patients undergoing free flap reconstructive surgery for head and neck cancer. (**a**) Schematic diagram of the study protocol. (**b**) Langendorff rat heart ischemia/reperfusion injury model using plasma dialysate from patients received remote ischemic conditioning or sham. (**c**) Protocol of ischemia/reperfusion injury and measurement of infarct size of the rat heart. *RIC* remote ischemic conditioning, *KHB* Krebs–Henseleit buffer.

The RIC protocol consisted of four cycles of 5-min ischemia/5-min reperfusion, induced by inflation of a pneumatic cuff up to 200 mmHg followed by deflation, applied to an upper or lower extremity contralateral side from the donor site. During sham-RIC, the cuff pressure was maintained at < 10 mmHg. The RIC or sham-RIC was applied twice: after anesthesia induction and at the end of surgery (Fig. 2a).

Tissue oxygen saturation (StO_2_) was measured using the InSpectra™ StO_2_ sensor (model 1615; Hutchinson Technology Inc., Hutchinson, MN, USA), using near-infrared spectroscopy (NIRS). The surface temperature of the tissue and the adjacent area (5 cm apart from the margin) was measured using an infrared thermometer (Non-Contact Thermometer, model NT1, AViTA Corporation, New Taipei City, Taiwan). Flap StO_2_ and the surface temperature of the flap and the adjacent area were measured before induction of anesthesia and on the first postoperative day. The room temperature was maintained at 24 ± 1 °C.

### Langendorff rat heart ischemia and reperfusion injury model

To prepare plasma dialysate, 30 mL of blood were obtained from five patients in each group^12^ at baseline and at the end of surgery, respectively (Fig. 2a). The blood was immediately centrifuged at 3000 rpm for 20 min, and the plasma fraction was separated to be dialyzed across a 12–14 kDa dialysis tubing membrane (Spectra/Por, Spectrum Laboratories, Inc., Rancho Dominguez, CA, USA) against a 20-fold volume of modified Krebs–Henseleit buffer (KHB) for 24 h at 4 °C (Fig. 2b). Modified KHB consisted of 118 mM NaCl, 4.7 mM KCl, 1.1 mM MgSO_4_·7H_2_O, 1.2 mM KH_2_PO_4_, and 1.8 mM CaCl_2_·2H_2_O^13^. To perfuse to the rat heart, the dialysate was supplemented with 25 mM NaHCO_3_ and 11 mM D-glucose and gassed with 95% O_2_/5% CO_2_ mixture, then the pH was adjusted to 7.4 at 37 °C^14^.

Male Sprague–Dawley rats, aged 9–12 weeks, were used in the Langendorff I/R injury model^14^. All animals were purchased commercially (KOATECH Corp.; Pyeongtaek-si, Gyeonggi-do, South Korea) and cared for in accordance with the Guidelines for Care and Use of Laboratory Animals issued by the IACUC of Seoul National University. Rats were anesthetized with inhalation of 6–8 vol% sevoflurane (Sojourn, Piramal Critical Care Inc., Bethlehem, PA, USA) and heparinized with intravenous administration of heparin (100 IU/kg) via a lateral tail vein. Following thoracotomy, the heart was excised and mounted on the Langendorff apparatus to be perfused with KHB solution in a retrograde manner^13^. Rats were euthanized by exsanguination after excision of the hearts under anesthesia.

After stabilization, the hearts were perfused with plasma dialysate for 15 min and then washed out before being subjected to 30-min global normothermic ischemia and the subsequent 60-min reperfusion (Fig. 2c)^14^. Constant flow with flow rate of 7–10 mL/min using peristaltic pump was used to perfuse the isolated rat hearts. KHB solution consisted of 118 mM NaCl, 25 mM NaHCO_3_, 11 mM D-glucose, 4.7 mM KCl, 1.1 mM MgSO_4_·7H_2_O, 1.2 mM KH_2_PO_4_, and 1.8 mM CaCl_2_·2H_2_O, was gassed with 95% O_2_/5% CO_2_ mixture, and adjusted to pH 7.4 prior to perfusion. The temperature of the hearts was maintained at 37℃ throughout the experiment.

Following reperfusion, the hearts were kept at − 20 °C for 30 min and then cut transversely into 5–6 slices of 1–2 mm thickness. The slices were then immersed in 1% 2,3,5-triphenyltetrazolium chloride for 15 min to distinguish viable tissue (stained red) from infarcted tissue (pale white). The slices were fixed in 10% formalin to enhance contrast and then digitally scanned to measure planimetric areas using ImageJ software (ver. 1.51; NIH, USA) by a blinded researcher. The infarct size was calculated as a percentage of the infarcted area within the left ventricles. The protocol was considered failed, and the results were excluded from the analyses, if one of the following criteria were met: time to perfusion > 3 min, significant ventricular arrhythmia > 5 min, heart rate < 100 or > 400 /min, or unstable contractility. Contractility of the heart was determined by assessment of vigorous contraction of the left ventricle and by assuring adequate heart rate. Measurement of heart rate was achieved by calculation of contraction of the heart over a measured period of time by recording.

### Data collection

Baseline patient characteristics and perioperative variables were collected. The incidence of flap re-operation, wound exploration, the rate of flap survival, and total or partial flap loss were assessed during hospitalization.

### Study outcomes and sample size calculation

The primary endpoint of the study was postoperative StO_2_ of the flap between the groups. The secondary endpoints were changes in StO_2_ from baseline and postoperative surface temperature of the flap. The organ-protective effects of RIC were assessed by comparing the infarct sizes of the rat heart^15^.

We determined the sample size based on a previous study, in which the StO_2_ of the anterolateral thigh after RIC was 55 ± 12%^10^. To detect clinically relevant changes in the flap StO_2_ (20%) between the two groups, 23 patients per group was calculated for a significance level of 5% and power of 85%. We enrolled 25 patients in each group, assuming a 10% dropout rate.

For animal experiments, we calculated sample size based on a previous study, in which the baseline myocardial infarct size using plasma dialysate from surgical patients was 38.6 ± 3.6%^16^. Assuming that a 25% reduction in infarct size is significant, four subjects per group were required at an alpha error of 5% and a power of 85%. Therefore, we calculated that five patients would be required per group, considering a 15% dropout rate. Each rat heart was treated only with one certain plasma dialysate obtained before or after each treatment in one patient. Therefore, a total of 20 rat hearts are required for 10 patients selected for dialysate preparation.

### Statistical analyses

The normality of the data was tested using Kolmogorov–Smirnov test. The data are presented as mean (standard deviations), median (interquartile range) or number (proportions). StO_2_ and the temperature of the flap were compared using the independent *t* test based on the results of the normality test. Other continuous variables were compared using the independent *t* test or the Mann–Whitney *U* test according to the normality of the data. Non-continuous variables were compared using Pearson’s chi square test or Fisher’s exact test. All analyses were performed in an intention-to-treat manner. IBM SPSS Statistics 21.0 (IBM Corp., Armonk, NY, USA) was used for the analyses. A *P* value < 0.05 was taken to indicate statistical significance.

## Results

Among 79 patients screened, 29 were excluded due to body mass index < 18 kg/m^2^ (n = 5), uncontrolled diabetes (n = 7), use of beta-blockers (n = 8), presence of peripheral vasculopathy (n = 2), previous radiation therapy (n = 4), and refusal to participate (n = 3) (Fig. 1). Then, 50 patients were randomized to receive either RIC or sham-RIC (each n = 25) between April 13, 2018 and July 8, 2019. After enrollment, four patients were excluded due to cancellation of the flap procedure, and 23 patients in each group were included in the analysis (Fig. 1).

The baseline characteristics of all included patients and selected patients for dialysate preparation are presented in Tables 1 and 2. The most frequent diagnosis was tongue cancer (17/46), and the most common donor site was anterolateral thigh (36/46, Table 1). Baseline StO_2_ and the surface temperature of the donor flap sites were comparable between the two groups (75 ± 19% vs 75 ± 17% and 35.8 ± 0.6 °C vs 36.0 ± 0.6 °C in the RIC vs sham-RIC groups; *P* = 0.987 and 0.221, respectively).

**Table 1:** Baseline characteristics of enrolled patients received remote ischemic conditioning or sham during free flap reconstructive surgery for head and neck cancer.

	RIC (n = 23)	Sham-RIC (n = 23)
Age, year	61 ± 14	60 ± 15
Male	15 (65.2%)	15 (65.2%)
Body mass index, kg/m^2^	22.0 (19.1–24.7)	21.5 (20.4–23.7)
**ASA physical status**		
II	19 (82.6%)	21 (91.3%)
III	4 (17.4%)	2 (8.7%)
Hypertension	7 (30.4%)	10 (43.5%)
Diabetes mellitus	5 (21.7%)	2 (8.7%)
**Smoking status**		
Never smoker	17 (74%)	19 (83%)
Current smoker	4 (17%)	3 (13%)
Previous smoker	2 (9%)	1 (4%)
**Diagnosis**		
Tongue cancer	10 (43.5%)	7 (30.4%)
Buccal mucosal cancer	1 (4.3%)	2 (8.7%)
Nasal cavity cancer	1 (4.3%)	1 (4.3%)
Maxillary sinus cancer	2 (8.7%)	4 (17.4%)
Palate cancer	3 (13.0%)	3 (13.0%)
Pyriform sinus cancer	0 (0.0%)	1 (4.3%)
Oropharyngeal cancer	5 (21.7%)	1 (4.3%)
Thyroid cancer	1 (4.3%)	1 (4.3%)
Other oral cavity cancer	0 (0.0%)	3 (13.0%)
**Flap site**		
Anterolateral thigh	20 (87.0%)	16 (69.6%)
Radial forearm	2 (8.7%)	2 (8.7%)
Latissimus dorsi	1 (4.3%)	2 (8.7%)
Fibular	0 (0.0%)	3 (13.0%)

Values are mean ± SD, number (proportion), or median (IQR).

*RIC* remote ischemic conditioning, *ASA* American society of anesthesiologist.

**Table 2:** Characteristics of patients selected for dialysate preparation for Langendorff rat heart experiments.

	RIC (n = 5)	Sham-RIC (n = 5)	*P* value*
Hypertension	0 (0%)	3 (60%)	0.167
Diabetes mellitus	1 (20%)	1 (20%)	> 0.999
**Smoking status**			> 0.999
Never smoker	4 (80%)	5 (100%)	
Current smoker	0 (0%)	0 (0%)	
Previous smoker	1 (20%)	0 (0%)	
**Location of RIC stimulus**			
Upper arm	5 (100%)	5 (100%)	

Values are number (proportion).

RIC, remote ischemic conditioning.

*P value between the RIC and sham-RIC groups.

**Figure 3:**
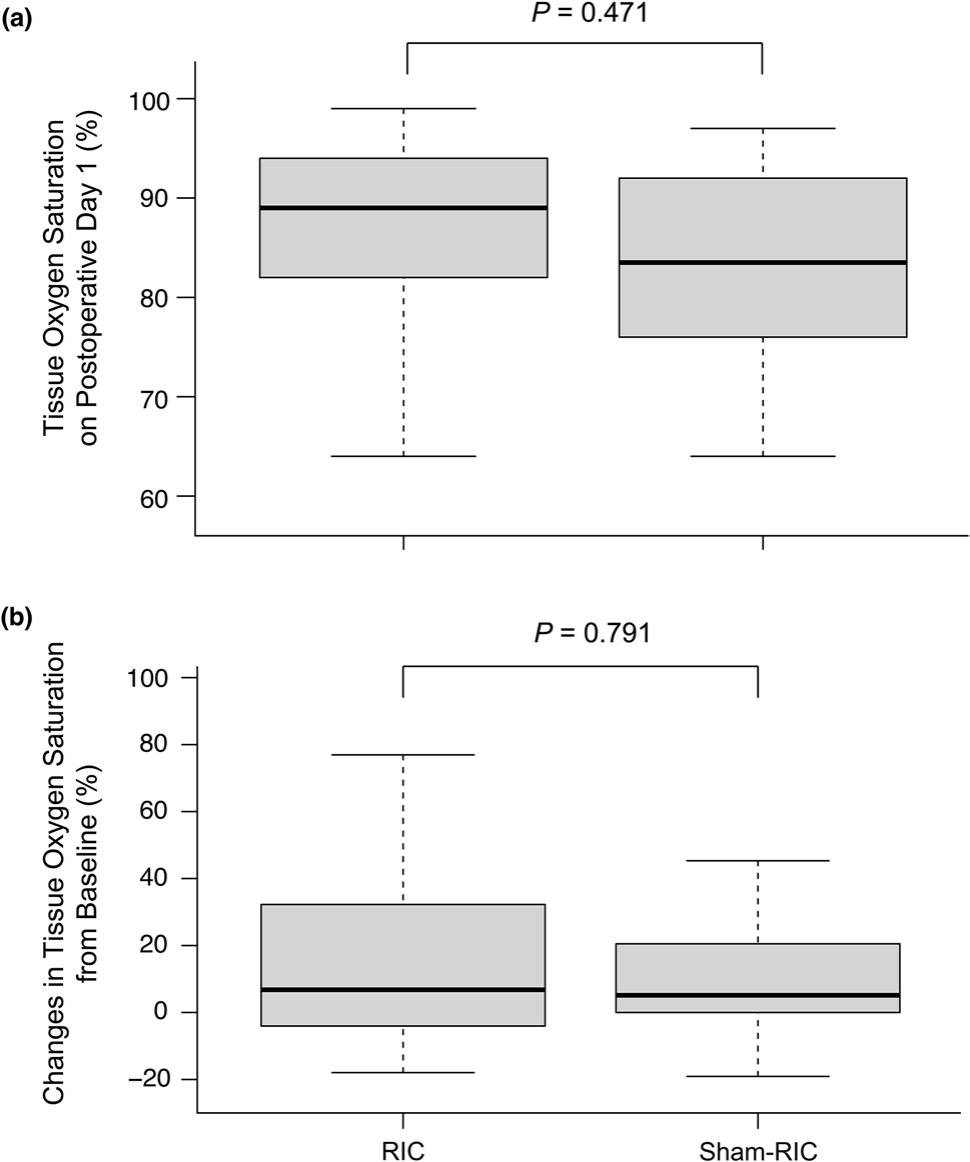
Boxplot of **(a)** tissue oxygen saturation of the flap on postoperative day 1 and **(b)** changes in tissue oxygen saturation of the flap site after free flap reconstruction compared to the baseline in the RIC and sham-RIC group. Horizontal line within the box indicates median values; lower and upper boundaries of the box indicate 25th and 75th percentiles, respectively; horizontal lines outside the box indicate the minimum and the maximum values of the data, respectively. Extreme outliers were omitted. *RIC* remote ischemic conditioning.

The primary endpoint, StO_2_ of the flap site on the first postoperative day was similar between the two groups (85 ± 12% vs 83 ± 9% in the RIC vs sham-RIC groups, respectively; *P* = 0.471; Fig. 3a). Changes in StO_2_ of the flap site compared to the baseline did not differ between the groups (*P* = 0.791; Fig. 3b). The surface temperature of the flap site on the first postoperative day was lower in the RIC group (36.9 ± 1.0 °C vs 37.5 ± 0.8 °C; *P* = 0.046), whereas the temperature of the adjacent area was comparable between the groups (36.9 ± 0.8 °C vs 37.3 ± 0.7 °C; *P* = 0.091). The difference in the surface temperature of the flap and the adjacent area did not differ between the groups (0.1 ± 0.5 °C vs 0.2 ± 0.5 °C in the RIC vs sham-RIC groups; *P* = 0.358).

**Figure 4:**
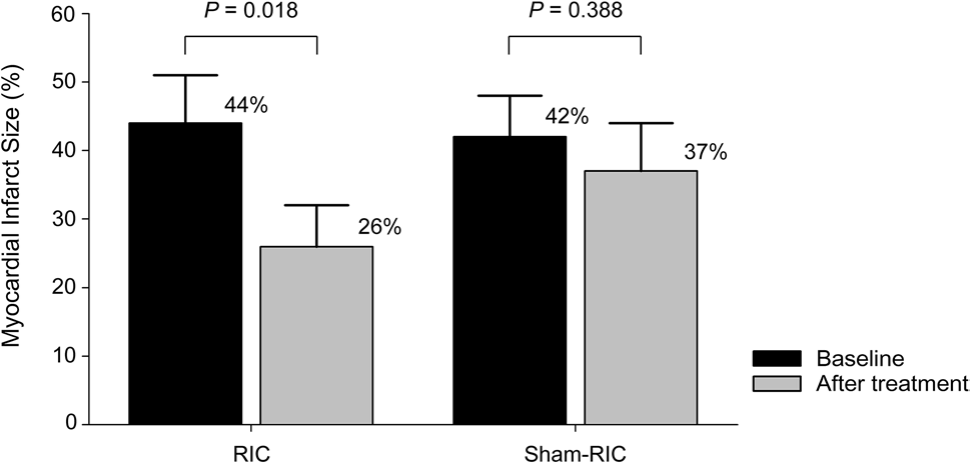
Infarct size of the rat hearts perfused with serum dialysate from patients before and after remote ischemic conditioning or sham treatment. *RIC* remote ischemic conditioning.

A total of 20 rat hearts were used and included in the analysis for animal experiments. The heart rate of the rat hearts was 265 ± 32 and 190 ± 21/min versus 252 ± 26 and 187 ± 10 /min at baseline and after reperfusion in the RIC versus sham-RIC groups (*P* = 0.517 and 0.804 between the groups). The infarct size of the rat hearts was significantly reduced after treatment of RIC dialysate compared to sham-RIC dialysate (44 ± 7% at baseline to 26 ± 6% after treatment versus 42 ± 6% to 37 ± 7%; *P* = 0.018 versus 0.388 in the RIC vs sham-RIC groups, respectively) (Fig. 4).

The rate of overall flap survival was 91.3%. The median StO_2_ of the flap on the first postoperative day was higher in the survived flaps (86%) compared to the failed flaps (67%), without significant difference (*P* = 0.299). Flap re-operation and wound exploration was required in two patients (8.7%) in each group (Table 3). There was no difference in flap loss rate, length of the intensive care unit and postoperative hospital stay between the groups (Table 3).

**Table 3:** Perioperative variables in patients received remote ischemic conditioning or sham during free flap reconstructive surgery for head and neck cancer.

	RIC (n = 23)	Sham-RIC (n = 23)	*P* value *
**Intraoperative variables**			
Duration of operation, min	420 (350–475)	440 (358–573)	0.510
Duration of anesthesia, min	455 (410–530)	495 (415–665)	0.583
Estimated blood loss, mL	580 (330–925)	630 (375–870)	0.545
RBC transfusion, unit	0 (0–0)	0 (0–0)	0.200
Infused crystalloid, mL	3300 (2450–5100)	3400 (2650–4500)	0.965
Urinary output, mL	860 (530–1295)	960 (570–1695)	0.374
**Location of RIC stimulus**			> 0.999
Upper arm	20 (87.0%)	19 (82.6%)	
Lower leg	3 (13.0%)	4 (17.4%)	
Flap re-operation	2 (8.7%)	2 (8.7%)	> 0.999
Wound exploration	2 (8.7%)	2 (8.7%)	> 0.999
Flap survival	21 (91.3%)	21 (91.3%)	> 0.999
**Flap loss**			0.350
Total flap loss	2 (8.7%)	2 (8.7%)	
Partial flap loss	0 (0.0%)	2 (8.7%)	
Peak hs-CRP, mg/dL	15.38 ± 4.51	12.91 ± 5.68	0.109
Peak lactic acid, mmol/L	2.97 ± 1.49	2.44 ± 1.55	0.247
Length of intensive care unit stay, h	17 (14–19)	16 (13–19)	0.748
Length of postoperative hospital stay, days	17 (15–21)	18 (14–22)	0.869

Values are median (IQR), number (proportion), or mean ± SD.

*RIC* remote ischemic conditioning, *RBC* red blood cell, *hs-CRP* high sensitivity C-reactive protein.

*P value between the RIC and sham-RIC groups.

## Discussion

In this study, RIC during free flap reconstructive surgery did not improve flap tissue oxygenation compared to sham-RIC. The surface temperature of the flap was lower in the RIC group compared to the sham-RIC group, without clinical significance. The myocardial infarct size in the experimental model was significantly reduced by treatment with RIC dialysate compared to sham-RIC dialysate.

The concept of ischemic conditioning was first described by Murry et al. in an experimental study where brief episodes of ischemia and reperfusion before subsequent ischemic insult significantly reduced infarct size in the canine myocardium^17^. Moreover, conditioning also attenuated I/R injury at remote sites apart from the target organ^18^. These protective effects seem to be associated with a reduction of muscle-ATP depletion and have energy-sparing actions during subsequent sustained ischemia^17,19^.

Based on the above, RIC is considered a simple, non-pharmacologic, and noninvasive strategy to improve flap survival. Indeed, several experimental studies have confirmed that survival of the transferred tissue improved with RIC in animal models^19–21^. In pigs, RIC reduced the infarct area of the latissimus dorsi, gracilis, and rectus abdominis muscle flaps following sustained I/R injury^19^. A single cycle of RIC via tourniquet on the hindlimb before flap dissection also enhanced survival of the adipocutaneous flaps in rats^20^. Moreover, the combination of pre- and post-conditioning reduced the necrotic area in skin flaps in rats^21^. In clinical studies, RIC improved microcirculation, as measured using laser doppler spectrophotometry of the anterolateral thigh donor site in healthy volunteers^22^. RIC of the surgical patients’ upper extremities also improved the microcirculation of various surgical flaps postoperatively^23^. RIC on the upper extremity induced more improved cutaneous blood flow of the anterolateral thigh compared to that on the lower extremity^22^. In our study cohort, the majority of the case received upper arm RIC mainly due to the need for preservation of both legs as potential donor sites.

In previous literature, the most common cause of total flap necrosis or urgent surgical reexploration after head and neck reconstructive surgery was arterial or venous thrombosis^1,3^. RIC delayed thrombotic occlusion in patients undergoing primary coronary intervention, suggesting beneficial effects on inhibition of thrombosis by reducing platelet reactivity^24^. Specific platelet P2Y_12_ receptor antagonists also induced cardioprotection beyond the inhibition of platelet aggregation^25,26^. Inhibition of platelet activation and thrombus formation might have beneficial effects during microvascular surgery with potential high risk of thrombotic complication.

Owing to the complex anatomy and physiology of the head and neck region, achieving flap viability after microvascular reconstructive surgery is challenging. Along with the subjective observations of the flap color or capillary refill, many objective approaches, such as doppler flowmetry, surface temperature, and NIRS^2^, can also be utilized to detect vascular thrombosis or a failing flap. Monitoring StO_2_ using NIRS is useful for detecting early arterial or venous thrombosis, and is related to improved flap survival^27,28^. Decreased StO_2_ was associated with vascular thrombosis and compromised flap vascularity^29,30^. Monitoring of StO_2_ using InSpectra™ sensor reliably reflected tissue ischemia caused by vascular occlusion^31^ and detected increase in lactate level earlier than cardiac index, mixed venous saturation, and mean arterial pressure^32^. The surface temperature has been suggested to reflect flap circulation and have shown significant differences in temperatures between the surviving and failing flaps^33,34^. On the contrary, other studies have found that surface temperature changes in flaps did not predict vascular compromise^35^.

In this study, we evaluated the effects of RIC, which was performed as a combination of pre- and post-conditioning (applied before and after free tissue transfer), on postoperative tissue oxygenation of the flap. Although we did not observe clinical benefits of RIC on flap oxygenation, organ-protective effects of RIC were demonstrated in the ex vivo myocardial I/R injury experiment. We thus confirmed RIC’s humoral transfer of cardioprotection with plasma or its derivatives to isolated perfused hearts as demonstrated in previous studies^36–40^.

One of major confounder of protective effects of RIC is the use of propofol during cardiovascular surgery^41^. In our previous study, RIC’s humoral transfer of cardioprotection was abrogated under propofol anesthesia, while it was effective in patients without any anesthetics^16^. The major methodological difference is that RIC was performed during continuous infusion of propofol in the previous study^16^, while propofol was only administered as a bolus at least 30 min prior to the first cycle of the RIC protocol in the current trial. Additionally, RIC stimulus was provided twice, as pre- and post-conditioning, at upper or lower extremity, which might have mass effects with potential dose–response relationship that the greater tissue mass would have released cardioprotective factors despite the use of propofol^42^. Moreover, we cannot exclude any possible mechanism related to the receptor organ rather than the effector organ that has not been exposed to propofol during experimental protocol.

Regarding StO_2_ measures, contrary to the results of improved superficial microcirculation up to 4 h after 3 or 5 cycles of RIC in a previous study^43^, StO_2_ of the flap measured on the first postoperative day was not affected by RIC, which was performed intraoperatively in this study. We chose the current RIC protocol, consisted of 4 cycles of ischemia and reperfusion, based on previous literature^12,44^ and feasibility to our anesthetized patients for the present study. The number of RIC cycles or timing of StO_2_ measurement from the completion of RIC might have influenced our results.

Myocardial injury after non-cardiac surgery is a serious complication and associated with poor prognosis, even without ischemic symptoms^45^. The mechanism of myocardial injury after non-cardiac surgery involves oxygen supply–demand mismatch along with anemia, hypoxemia, and hypotension^46^. However, it is not always feasible to detect myocardial injury based on the serial measurements of cardiac biomarkers or electrocardiographic findings after non-cardiac surgery. Nevertheless, it is important to prevent potential myocardium injury or infarction during non-cardiac surgeries. Cardioprotective effects of RIC have been investigated extensively in the areas of cardiovascular disease^47,48^. Although long-term clinical benefits have not been proven^49^, RIC appears to have a protective role against myocardial injury, even in non-cardiac procedures^50^. Consistently, we observed myocardial protection of RIC in this study.

This study had several limitations. First, there were differences in proportion of preoperative risk factors, which may act as confounding factors, between the groups (Table 1). Although the differences had no statistical significance (*P* = 0.359 for hypertension and *P* = 0.414 for diabetes mellitus), we cannot completely exclude any influence of these factors on our results as the number of included patients is low. Second, we included a relatively small number of patients with various reconstructive sites. Although the most common reconstructive site was the tongue (37%) and anterolateral thigh flap was used in 78%, heterogenous reconstructive sites might have affected any RIC effects. Moreover, although we observed higher postoperative StO_2_ in the survived flaps compared to the failed flaps (86% vs 67%), any comparison between the survived and failed flaps is not adequately powered in the current analysis. Therefore, further studies are required to assess the relationship between StO_2_ and the viability of the flap. Third, we had a small proportion of patients who were at high risk of flap complications, such as currently smoking or obese patients. We also excluded patients who underwent preoperative radiation therapy. Considering RIC had organ-protective effects in an experimental model, RIC might be helpful in patients with higher risk of flap malperfusion. Further studies are needed to investigate the effects of RIC in high-risk surgical patients. Lastly, we did not directly measure the coronary flow or ventricular function during the animal experiments. However, the experiments followed validated protocol in accordance with previous literature with predefined criteria, and acceptable infarct sizes following I/R injury were obtained initially in both groups.

In conclusion, RIC did not improve flap tissue oxygenation after head and neck cancer reconstructive surgery. There was evidence of a myocardial-protective role of RIC in the ex vivo I/R injury model using plasma dialysate from patients.

## Data Availability

The datasets analyzed during the current study are available from the corresponding author on reasonable request.

## Abbreviations

I/R: Ischemia/reperfusion
RIC: Remote ischemic conditioning
IACUC: Institutional Animal Care and Use Committee
NIH: National Institutes of Health
StO_2_: Tissue oxygen saturation
NIRS: Near-infrared spectroscopy
KHB: Krebs–Henseleit buffer
